# Obituary

**Published:** 1867-12

**Authors:** 


### *Obituary.*

Jesse Judkins, M.D., Prof. of Anatomy and Surgical Pathology, in the Miami Medical College, Cincinnati, died, after a lingering illness, in that city, a few days since. He had acquired high distinction in the professioral chair—had acquired a large practice, and the personal respect and esteem of all who knew him. We suppose him to have been about fifty years of age. By his decease the Medical Profession have suffered a loss which cannot easily be repaired.

